# Antifungal and Anti-Biofilm Activities of Acetone Lichen Extracts against *Candida albicans*

**DOI:** 10.3390/molecules22040651

**Published:** 2017-04-19

**Authors:** Marion Millot, Marion Girardot, Lucile Dutreix, Lengo Mambu, Christine Imbert

**Affiliations:** 1 Laboratoire de Chimie des Substances Naturelles, Faculté de Pharmacie, 2 rue du Dr Marcland, 87025 Limoges, France; lengo.mambu@unilim.fr; 2 Laboratoire Ecologie et Biologie des Interactions, Equipe Microbiologie de l’Eau, Université de Poitiers, UMR CNRS 7267, F-86073 Poitiers, France; marion.girardot@univ-poitiers.fr (M.G.); lucile.dutreix@yahoo.fr (L.D.); christine.imbert@univ-poitiers.fr (C.I.)

**Keywords:** lichens, biofilm, *Candida albicans*, screening, chemical profiling

## Abstract

*Candida albicans* is a commensal coloniser of the human gastrointestinal tract and an opportunistic pathogen, especially thanks to its capacity to form biofilms. This lifestyle is frequently involved in infections and increases the yeast resistance to antimicrobials and immune defenses. In this context, 38 lichen acetone extracts have been prepared and evaluated for their activity against *C. albicans* planktonic and sessile cells. Minimum inhibitory concentrations of extracts (MICs) were determined using the broth microdilution method. Anti-biofilm activity was evaluated using tetrazolium salt (XTT) assay as the ability to inhibit the maturation phase (anti-maturation) or to eradicate a preformed 24 h old biofilm (anti-biofilm). While none of the extracts were active against planktonic cells, biofilm maturation was limited by 11 of the tested extracts. Seven extracts displayed both anti-maturation and anti-biofilm activities (half maximal inhibitory concentrations IC_50_mat_ and IC_50_biof_ ≤ 100 µg/mL); *Evernia prunastri* and *Ramalina fastigiata* were the most promising lichens (IC_50_mat_ < 4 µg/mL and IC_50_biof_ < 10 µg/mL). Chemical profiles of the active extracts performed by thin layer chromatography (TLC) and high performance liquid chromatography (HPLC) have been analyzed. Depsides, which were present in large amounts in the most active extracts, could be involved in anti-biofilm activities. This work confirmed that lichens represent a reservoir of compounds with anti-biofilm potential.

## 1. Introduction

Lichens are symbiotic organisms resulting from the association between a fungus, called the mycobiont (usually an ascomycete), and photoautotrophic partners, green algae, and/or cyanobacteria, called the photobiont. Recently, a third partner embedded in the lichen cortex was identified as a basidiomycete yeast [1]. The lichen thallus is a support for other microorganisms living inside and outside the thallus, including endo- and epi-lichenic fungi and bacteria [2,3]. Interactions exist within this complex ecosystem and many of the molecules that make up this chemical environment are involved in interactions between the community members influencing overall community homeostasis and survival. This complex community is a potential source of new pharmaceutical drugs [4,5].

*Candida* spp. belongs to the healthy human microbiota and *Candida albicans* is a normal inhabitant of the gastrointestinal tract, reproductive tract, oral cavity, and skin of most humans [6,7]. *C. albicans* is also an opportunistic yeast causing superficial and invasive infections, especially among elderly and/or frail patients (immunocompromised, with long term stays in intensive care units, after digestive surgery, etc.) [8,9,10]. Oral candidiasis is a common superficial and opportunistic infection accounting for significant morbidity, especially in immunocompromised patients and, more generally, in denture wearers [11,12,13]. Biofilms may form on a variety of surfaces, with catheter, dental, and mucosal surfaces being the most common. Adherence of *Candida* yeasts to these host surfaces is a prerequisite for subsequent colonization and biofilm formation [14,15,16] and, therefore, is a key step in the process leading to oral candidiasis. The adhesion phase is followed by the maturation of the biofilm, characterized by hyphae and extracellular matrix production [17]. Biofilm formation is implicated in 80% of infections, and is an important virulence factor as sessile cells become poorly or not susceptible to both antimicrobials and host immune responses [18]. 

Natural compounds, such as polyphenols, have begun to be tested against biofilms. Good activities have been described for curcumin and pyrogallol against *C. albicans* biofilm [19]. Purpurin, a natural anthraquinone pigment, has demonstrated an effect against *C. albicans* hyphal formation and biofilm development [20]. Lichen extracts and their metabolites have been widely studied for their antimicrobial properties, but their anti-biofilm potential is still poorly explored. Mitrovic et al. have studied the anti-biofilm activity of two common lichens (*Platismatia glauca* and *Pseudevernia furfuracea*) against *S. aureus* biofilms. The acetone, ethyl acetate or methanol extracts showed activities with a biofilm inhibitory concentration (BIC) ranging from 0.63 mg/mL to 1.25 mg/mL [21]. While few lichen metabolites have been evaluated against *C. albicans* biofilms, no lichen extracts have been tested for such an activity. Chang et al. have demonstrated the anti-biofilm activity of retigeric acid B, a pentacyclic triterpenoid isolated from the lichen *Lobaria kurokawae*, by inhibiting *C. albicans* yeast-to-hypha transition in infected mice and by acting synergistically with azoles to block *C. albicans* biofilm formation [22].

Thus, lichens appear to be a non-negligible source of bacterial or fungal anti-biofilm compounds. In this study, a screening of 38 acetone extracts, obtained from lichens mostly collected in Limousin (France), was performed against planktonic and sessile *C. albicans* cells. Lichens selected for this work belonged to nine different families (Parmeliaceae and Cladoniaceae, principally). Samples have been collected on trees, rocks, or soil, implying variations on their ecological environment (insects, herbivores, fungi, bacteria). Their anti-biofilm activities, as well as their chemical composition, were investigated.

The aim of this study was, thus, to investigate lichens from the center-west of France as new natural sources for anti-biofilm agents and, in particular, to search for lichen extracts able to both inhibit the maturation of *C. albicans* biofilms (suggesting a prophylactic interest) and reduce an already formed biofilm of *C. albicans* (suggesting a curative interest).

## 2. Results and Discussion

### 2.1. Preparation of Extracts

Extracts were obtained by the maceration of the lichen thallus in acetone, at room temperature. This extraction protocol led to good extraction rates, ranging between 0.2% and 10.5% for the majority of lichens (see Table 1), and to a wide diversity of secondary metabolites (terpenes, sterols, depsidones, depsides, dibenzofurans, xanthones). Furthermore, the extraction of primary metabolites, such as sugars, as well as thermal degradation of thermolabile compounds (depsides or diphenylethers) were limited.

Extracts have been analyzed by thin layer chromatography (TLC) in two system solvents and observed under ultraviolet (UV) and after spraying anisaldehyde sulfuric reagent and/or by high performance liquid chromatography (HPLC). A comparison with standards and bibliographic data allowed the identification of major compounds in almost all extracts [23,24,25]. Polyphenolic compounds (depsides, depsidones, dibenzofurans, and xanthones) and terpenoids predominated in the chemical profiles of the studied lichen extracts, with often one or two major metabolites (Table 1 and Supplemental Figure S1).

### 2.2. Antifungal Activity

The 38 lichen extracts were first evaluated for their activity against *C. albicans* planktonic yeast. None of the extracts displayed antifungal activity against these cells with minimal inhibitory concentrations (MICs) > 100 µg/mL (fluconazole MIC: 4 µg/mL). These results are consistent with data reported in the literature, which suggest poor activities of some lichen extracts and their compounds against planktonic microbes, and especially towards *C. albicans*, with MICs being often above 500 µg/mL [21,26,27].

### 2.3. Activity against Biofilm Maturation

Extracts were then tested against the growth of *C. albicans* biofilms on polystyrene substrates (anti-maturation activity) by incubating with very young biofilms (2 h adhered cells) for 24 h or 48 h. The half maximal inhibitory concentrations (IC_50_) of the extracts are presented in Table 2. Eleven extracts significantly inhibited biofilm growth onto polystyrene surfaces (1.5 µg/mL ≤ IC_50_mat 48h_ ≤ 100 µg/mL; *p* < 0.03) after a 48 h incubation, suggesting their ability to delay biofilm maturation. 

Four extracts (*Anaptychia ciliaris*, *Cetraria islandica*, *Peltigera rufescens*, and *Xanthoria parietina*) were not promising as anti-maturation agents due to the lack of activity at 24 h and their low activity at 48 h and were not selected for further investigations.

Among the active extracts, only four (*Evernia prunastri*, *Peltigera hymenina*, *Ramalina fastigiata*, and *Xanthoparmelia conspersa*) were active after both 24 h and 48 h incubation time, but the IC_50_ obtained after 24 h were always higher (25 µg/mL ≤ IC_50_mat 24h_ ≤ 100 µg/mL) than those observed after 48 h (1.5 µg/mL ≤ IC_50_mat 48h_ ≤ 25 µg/mL). This observation suggests that an extended contact between extracts and microbes is needed to obtain overall inhibitory activity. Additionally, it also suggests that these extracts could be promising for long-term prophylactic action. It is noteworthy that *E. prunastri* and *R. fastigiata* were the most active extracts against maturation phase, with IC_50_mat 24h_ = 25 µg/mL and IC_50_mat 48h_ ≤ 3.1 µg/mL.

### 2.4. Anti-Biofilm Activity

Lichen extracts which demonstrated an anti-maturation activity with IC_50_mat 48h_ ≤ 50 µg/mL have been selected and tested against a pre-formed *C. albicans* biofilm 24 h of age by incubation for 48 h (according to the previous observation with anti-maturation tests), to evaluate their interest for a curative approach. Two complementary methods were used, tetrazolium salt (XTT) assay and colony-forming unit (CFU) counts. The IC_50_ and percentage of inhibition of the extracts are presented in Table 2. The seven tested extracts demonstrated significant anti-biofilm activity (≤10 µg/mL ≤ IC_50_biof_ ≤ 100 µg/mL; *p* < 0.0002).

Four extracts, *C. uncialis*, *E. prunastri*, *R. fastigiata*, and *X. conspersa* showed the highest activity with IC_50_biof_ ≤ 10 µg/mL. CFU counts confirmed this anti-biofilm activity by demonstrating a significant decrease in the yeast community constituting the biofilm (inhibition ≥ 80%; *p* < 0.04) (Table 2). This decrease was also confirmed by direct observations (inverted optical microscopy) (data not shown). This multi-method approach makes it possible to be sure that there is no interference between lichen extracts and XTT, leading to false positives. 

Additionally, microscopic observations performed at the end of anti-biofilm tests using trypan blue staining showed that yeasts growing as biofilm were still alive after treatment (all cells were colorless), suggesting a non-lethal effect of the active extracts. Thus, these preliminary observations and results suggest that active extracts may act thanks to a dispersant or removing action that would not disrupt the fungal cell membrane. 

The anti-biofilm activity of the studied lichens is poorly documented and no bibliographic data reported anti-biofilm activity for the tested extracts. Thus, results obtained for the seven lichens whose extracts have demonstrated anti-maturation and anti-biofilm activities are innovative in this field of research.

### 2.5. Comparison of Chemical Profiles of Active and Inactive Extracts

Regarding the compounds in active and inactive extracts, some metabolites did not appear to be involved in the anti-maturation effect observed. Thus, the dibenzofurans placodiolic, pannaric, or didymic acids (predominant in *Leprocaulon microscopicum*, *Lepraria membranacea*, and *Cladonia incrassate*, respectively), the depsides atranorin and thamnolic acid (predominant in *Pseudevernia furfuracea* and *Cetrelia olivetorum* for atranorin, and predominant in *Cladonia parasitica, C. squamosal*, and *Usnea florida* for thamnolic acid), the depsides with aliphatic chains divaricatic and perlatolic acids (present in *Neofuscellia pulla*); the tridepsides gyrophoric acid and tenuiorin (predominant in *Lasallia pustulata* and *Peltigera* species), the depsidone norstictic acid (predominant in *Pleurosticta acetabulum*) or the aliphatic acid caperatic acid (predominant in *Flavoparmelia caperata* and *Platismatia glauca*) and rocellic acid (*Lepraria membranacea*), and the triterpene zeorin (present in *Leprocaulon microscopicum*) were reported in inactive extracts. Lichens with a chemical profile dominated by terpenoids, (in the genus *Peltigera*), were also mostly inactive.

Some compounds (such as usnic, fumarprotocetraric, protocetraric, salazinic, and squamatic acids), were present both in active and inactive extracts. Their role in the biological activity is more questionable. Focusing on the widespread dibenzofuran, usnic acid, a weak activity has been shown for lichens containing high proportions of this metabolite. This observation was confirmed by a HPLC dosage using a calibration curve with usnic acid (Supplemental Figure S2). Usnic acid represent 40% of the dried weight of the extract in *Usnea florida* and 20% of the dried weigh of the extract for *Flavoparmelia caperata* [28]. This result suggested the poor implication of this compound against the development and maturation of *Candida* biofilms. 

Finally, some compounds (stictic acid, evernic acid, parietin) were predominant only in active extracts (*X. conspersa*, *E. prunastri*, *R. fastigiata*, and *X. parietina*) suggesting their implication in the anti-maturation process of *C. albicans* biofilms (Supplemental Figure S1).

The extracts with the highest activity against both growing biofilms and 24 h old biofilms, *E. prunastri* and *R. fastigiata*, were dominated by the presence of the previously-mentioned evernic acid [29,30]. *E. prunastri* and evernic acid have already been studied for their antiproliferative and antimicrobial activities [31,32]. In a recent publication, the effect of evernic acid on bacterial biofilm has been evaluated and this depside has demonstrated a capacity to inhibit *Pseudomonas aeruginosa* quorum sensing systems [33].

Stictic acid, is a depsidone dominated the chemical composition of the extract of *X. conspersa* [34]. Antioxidant, antimicrobial, and apoptotic effect have already been described for stictic acid but no anti-biofilm activity is reported [35,36]. We suggested here the possible interest of this compound against *C. albicans* biofilms.

The other *Xanthoparmelia*, *X. tinctina*, has a complex chemical composition dominated by usnic acid and the depsidone salazinic acid. Surprisingly, salazinic acid is also present in lichens without any activity suggested possible synergistic mechanism inside this extract.

Another depside, squamatic acid, whose structure is closely related to evernic acid, is associated to usnic acid in *C. uncialis* extract ,and could also be involved in anti-biofilm activities [26]. The large proportion of (−)-usnic acid in this extract invites to distinguish the difference of activity between the two isomers. Studies reported activities of usnic acid against bacterial biofilms (*Staphylococcus*, *Streptococcus*, etc.) but also against fungal biofilms [37,38,39,40]. A recent study demonstrated that usnic acid exhibited a significant biofilm inhibition against azole-resistant and azole-sensitive *C. albicans* strains (71.08% and 87.84%, respectively). This effect would be mediated by oxidative and nitrosative stress, with a significant accumulation of intracellular and extracellular reactive oxygen species [41]. Usnic acid also inhibited the yeast to hyphal switch and reduced the thickness of matured biofilms of *C. albicans* [39]. Furthermore, it was able to reduce various sugars present in the exopolysaccharide layer. However, this compound does not appear to be effective against *C. krusei* biofilms and the effect is controversial against *C. parapsilosis* [38,42].

*P. hymenina* extract is constituted by a mixture of terpenoids and unidentified depsides and the role of each compound in the activity needs to be further established. The chemical profile of *C. ramulosa*, showed the presence of fumarprotocetraric acid along with another unidentified compound (Supplemental Figure S1).

Thus, this preliminary screening is a springboard towards additional investigations on lichen metabolites. Indeed, the pure compounds will be isolated in sufficient quantity from lichens and will be evaluated. If the activity against biofilms is not confirmed, synergistic effects and identification and isolation of minor compounds will be undertaken.

## 3. Experimental

### 3.1. Phytochemical Analysis

#### 3.1.1. Lichen Material

Lichens have mainly been collected from soil and trees in the countryside around Limoges city. Their identification was confirmed by lichenologists belonging to AFL (French Association of Lichenology). Voucher specimens were deposited at the herbarium of the faculty of pharmacy of Limoges. Place, date of collection, as well as reference numbers of the voucher specimens, are summarized in Supplemental Table S1.

#### 3.1.2. Extraction of Lichen and Analytical Studies

The dried thalli of lichens (5 g) were extracted with acetone (10 mL, three times) at room temperature. After filtration, the acetone filtrates were concentrated under reduced pressure to give solid extracts.

TLCs were performed on pre-coated silica gel aluminium sheets (Kieselgel 60 F_254_, 0.20 mm, Merck KGaA, Darmstadt, Germany). Extracts were dissolved in acetone (at a concentration of 5 mg/mL) before being deposited on the TLC sheets and eluted in two system solvents: *n*-hexane–ethylether–HCOOH 130:80:20 and toluene–EtOAc–HCOOH, 70:20:5. The visualization of the plates was carried out under UV light (254 and 365 nm). Then TLCs were sprayed with the anisaldehyde sulfuric reagent followed by heating at 100 °C. This reagent leads to purple spots for dibenzofurans, red spots for depsides, yellow and orange spots for depsidones, and pink and blue spots for terpenes. The following standards have been used: atranorin; zeorin; tenuiorin, evernic, gyrophoric, thamnolic, squamatic, norstictic, fumarprotocetraric acid, protocetraric, salazinic, stictic, didymic, pannaric, placodiolic, usnic, rocellic, and caperatic acids. The standards have been previously isolated in our laboratory from various lichen species and identified on the basis of NMR and MS spectra.

HPLC analyses of the active extracts were performed on a Waters Alliance 2690 (Waters Corporation, Milford, MA, USA) using a reversed-phase Hibar^®^ LiChrospher^®^ 100 C-18 column (5 µm, 250 × 4 mm, Merck KGaA, Darmstadt, Germany ) and using a photodiode-array detector (Waters 996). The elution solvent MeOH–H_2_O–H_3_PO_4_ 80:20:0.9 has been used on isocratic mode at a flow rate of 1 mL/min according to the protocol of Yoshimura et al. [43]. Lichen extracts were prepared at 5 mg/mL in a mixture of methanol and DMSO (90:10).

### 3.2. Biological Activities

#### 3.2.1. Minimal Inhibitory Concentration of Lichen Extracts

Stock solutions of lichen extracts were prepared in DMSO at 10 mg/mL and shaken. Their minimum inhibitory concentrations (MICs) were determined by a broth microdilution method in order to determine their antifungal activities (CLSI reference M27-A3 micromethod adapted protocol). The *Candida albicans* ATCC 3153 strain, purchased from the American Type Culture Collection, was used. Briefly, yeasts were first grown for 24 h on Sabouraud agar slants (Sanofi Diagnostics Pasteur, Marnes-la-Coquette, France) and *C. albicans* inocula were prepared by suspending yeasts in sterile physiological serum and adjusting to 0.5 MacFarland of absorbance. A one-thousandth dilution in RPMI (Roswell Park Memorial Institute)-MOPS (4-Morpholinepropanesulfonic acid) (Sigma, St. Louis, MO, USA) of this previous solution was made.

Serial two-fold dilutions of each stock solution were prepared in RPMI-MOPS in 96-well microtiter plates (Fisher Scientific, Illkirch, France) over the range 100 μg/mL to 0.048 μg/mL final (final DMSO concentrations did not exceed 2% of the overall volume in wells). The same volume per well of yeast culture was then added. Some wells were reserved for controls: non-treated yeasts (negative control); yeasts treated by DMSO 2%; and yeasts treated by fluconazole (positive control). The MICs were determined after incubation for 24 and 48 h at 37 °C without shaking. All tests were performed in duplicate in at least two separate experiments. Lichen extracts with a MIC less than 50 μg/mL were considered as potentially useful.

#### 3.2.2. Anti-Biofilm Tests

The tests were performed using the stock solutions of lichen extracts previously prepared. Serial two-fold dilutions of each stock solution were prepared in Yeast Nitrogen Base medium (Difco, Detroit, MI, USA), supplemented with 50 mM glucose (Sigma, St. Louis, MO, USA) (YNB-Glc) over the final range 200 μg/mL to 0.097 μg/mL for the anti-maturation test or at 100, 50, and 10 μg/mL for the anti-biofilm test (final DMSO concentrations did not exceed 2% of the overall volume in wells).

As for MIC assay, *C. albicans* ATCC 3153 yeasts were first grown for 24 h on Sabouraud agar slants. Then, four loopfuls of this culture were carefully transferred to 30 mL of YNB-Glc and incubated overnight at 37 °C without shaking. Obtained blastospores were harvested, washed twice in 0.1 M phosphate-buffered saline (PBS, pH 7.2), and adjusted to 1 × 10^7^ blastospores/mL in YNB-Glc.

Non-stimulated saliva collected from 12 healthy volunteers was pooled, altered with dithiothreitol (Sigma, St. Louis, MO, USA) (2.5 mmol/L), shaken on ice for 10 min, and centrifuged for 20 min at 3000× *g* and 4 °C (Cr3i centrifuge Jouan). The supernatant was diluted in three volumes of distilled water (25%), and filtered through a 0.45 μm membrane. This 25% sterile stock solution was stored at −80 °C.

Untreated 96-well tissue culture plates were filled with 200 μL of human saliva at 2%, obtained by dilution of the 25% sterile stock solution in distilled water, incubated for 1 h at 37 °C, and then completely aspirated; this pretreatment was done to encourage biofilm formation. Two-hundred microliters per well of yeast culture were then added. After 2 h of incubation at 37 °C for the anti-maturation test and 24 h of incubation at 37 °C for the anti-biofilm test, the non-adherent yeasts were removed by washing with PBS. Then, 250 μL of YNB-Glc and 50 μL of each dilution of lichen extracts were added to each well. Some wells were reserved for controls: non-treated yeasts (negative control) and yeasts treated by DMSO 2%. The plates were incubated for 24 h and/or 48 h at 37 °C. Then spent media and free-floating microorganisms were removed by aspiration. Wells were washed twice with PBS and observed under inverted optical microscope (IX51^®^ inverted microscope, Olympus America Inc., Melville, NY, USA) prior to biofilm quantification using a previously described metabolic assay based on the reduction of a tetrazolium salt (XTT) [44,45]. Briefly, 300 mg/L XTT (Sigma) and 0.13 mM menadione (Sigma) were added to 200 μL of PBS in each well. Plates were incubated for 3 h at 37 °C without shaking, then gently agitated and XTT formazan was measured colorimetrically at 450 nm (microplate reader LP400; Sanofi Diagnostics Pasteur) in order to determine the concentration inhibiting 50% of biofilm development. Background formazan values were determined with plates containing PBS only or containing PBS, XTT, and menadione; these values did not exceed 0.005 absorbance units and were, therefore, considered not significant. All anti-maturation and anti-biofilm tests were performed in duplicate and sextuplicate, respectively, in at least two separate experiments. 

Statistical analysis was conducted thanks to the Kruskal-wallis test to determine differences among the test groups [46].

#### 3.2.3. CFU Counts and Trypan Blue Staining Assays

At the end of anti-biofilm tests, four wells were used to conduct CFU counts: adherent cells treated or not with the highest concentration of lichen extract were scraped; the obtained cells were resuspended in PBS and sowed twice on Sabouraud agar plates after the dilution process. Colonies were counted after 24 h of incubation at 37 °C and the inhibition percentages ± standard deviations (SD) of cultivable cells were determined. Statistical analysis was conducted thanks to the Wilcoxon test [46].

Cell viability was evaluated thanks to trypan blue (Sigma, St. Louis, MO, USA) staining using four other wells. Briefly, the diluted dye was added to the cells, treated or not, and adhered to the bottom of the wells and living (colorless) and dead (blue) cells were counted under a microscope. Assays were repeated in at least two separate experiments.

## 4. Conclusions

Among the 38 extracts evaluated, seven of them demonstrated anti-maturation and anti-biofilm activities against *C. albicans* with IC_50_mat 48h_ and _biof_ ≤100 µg/mL. *E. prunastri* and *R. fastigiata* were the most promising lichens (IC_50_mat 48h_ < 4 µg/mL and IC_50_biof_ < 10 µg/mL). They exert on the mature biofilm a dispersant or removing action that would not disrupt the fungal cell membrane. The activities (against both growing biofilms and 24 h old biofilms) of the seven promising extracts will have to be confirmed by studying a large panel of clinical strains in order to check that these activities are not strain-dependent. These results complete the few available data concerning anti-biofilm interests of lichen extracts. For the first time this study underlines the ability of seven lichen species from Limousin to slow down the maturation phase of *C. albicans* biofilms (which is very important to avoid yeast dispersion) and to reduce already-formed biofilms (which contributes to reducing the infectious reservoir). The chemical investigation and comparisons performed on chemical profiles of active and inactive extracts underline that depsides (evernic and squamatic acids) and depsidones (stictic acid) may be involved in the anti-biofilm activity. The lichen compounds should now be isolated to be further investigated for their activities and possible synergistic actions.

## Figures and Tables

**Table 1:** Lichen species, extraction rates, and chemical composition of the lichens reported in the literature data and the main metabolites present in acetone extracts (determined by TLC and/or HPLC). Nd = non determined.

No.	Lichens	% Yield	Theoretical Chemical Composition of Lichen [25]	Main Metabolite(s) Present in the Acetone Extracts
**1**	*Anaptychia ciliaris*	0.2	Nd	Nd
**2**	*Bryoria fuscescens*	3.0	Cetraric acid; fumarprotocetraric acid	Fumarprotocetraric acid
**3**	*Cetraria islandica*	0.8	Protocetraric acid; protolichesterinic acid; fumarprotocetraric acid	Fumarprotocetraric acid
**4**	*Cetrelia olivetorum*	6.0	Atranorin; perlatolic acid; imbricaric acid and derivatives; fatty acids	Atranorin
**5**	*Cladonia fimbriata*	1.0	Atranorin; convirensic acid; fumarprotocetraric and protocetraric acids	Atranorin Fumarprotocetraric acid
**6**	*Cladonia furcata*	0.8	Atranorin; cetraric and fumarprotocetraric acids	Fumarprotocetraric acid
**7**	*Cladonia glauca*	1.9	Squamatic acid	Squamatic acid
**8**	*Cladonia gracilis*	1.6	Atranorin; fumarprotocetraric acid; quaesic acid	Fumarprotocetraric acid
**9**	*Cladonia incrassata*	5.5	(−)-Usnic acid; squamatic acid; didymic, subdidymic and condidymic acids	Didymic acid; squamatic acid
**10**	*Cladonia parasitica*	1.9	Barbatic acid; thamnolic acid and derivatives	Thamnolic acid
**11**	*Cladonia ramulosa*	4.6	Fumarprotocetraric acid and derivatives; protocetraric and sekikaic acids	Unidentified depside fumarprotocetraric acid
**12**	*Cladonia rangiferina*	1.5	Atranorin and fumarprotocetraric acid	Atranorin; fumarprotocetraric acid
**13**	*Cladonia scabriuscula*	2.0	Atranorin; fumarprotocetraric and protocetraric acids	Fumarprotocetraric acid
**14**	*Cladonia squamosa*	2.6	Barbatic acid; squamatic acid; thamnolic acid and derivatives; triterpenoids	Squamatic acid; thamnolic acid
**15**	*Cladonia subulata*	4.4	Fumarprotocetraric and quaesitic acids	Fumarprotocetraric acid
**16**	*Cladonia uncialis*	1.9	Squamatic acid and (−)-usnic acid	Squamatic acid and usnic acid
**17**	*Evernia prunastri*	7.5	Atranorin; chloroatranorin; evernic acid and (+)-usnic acid	Evernic and usnic acids
**18**	*Flavoparmelia caperata*	6.2	Atranorin; caperatic acid; protocetraric acid and (+)-usnic acid	Caperatic acid; protocetraric acid and usnic acid
**19**	*Hypogymnia physodes*	8.7	Atranorin; chloroatranorin; physodalic acid; physodic acid and derivatives; protocetraric acid	Fumarprotocetraric and other unidentified depsides or depsidones
**20**	*Lasallia pustulata*	5.8	Gyrophoric acid	Gyrophoric acid
**21**	*Lepraria membranacea*	5.6	Pannaric acid	Pannaric and rocellic acids
**22**	*Leprocaulon microscopicum*	8.4	Placodiolic acid; (−)-usnic acid; zeorin	Usnic acid; placodiolic acid and zeorin
**23**	*Nephroma parile*	2.8	Terpenoids	Terpenoids
**24**	*Neofuscellia pulla*	2.1	Divaricatic acid; perlatolic acid; stenosporic acid and derivatives; gyrophoric acid	Divaricatic and perlatolic acids
**25**	*Parmelia saxatilis*	10.1	Atranorin; chloroatranorin; salazinic and consalazinic acids	Salazinic acid
**26**	*Parmelia sulcata*	2.9	Atranorin; chloroatranorin; salazinic and derivatives; lobaric acid	Salazinic acid
**27**	*Platismatia glauca*	10.5	Atranorin; caperatic acid and pseudoplacodiolic acid	Atranorin; caperatic acid
**28**	*Peltigera collina*	1.4	Terpenoids	Tenuiorin
**29**	*Peltigera horizontalis*	0.7	Terpenoids	Tenuiorin and terpenoids
**30**	*Peltigera hymenina*	1.7	Gyrophoric acid and terpenoids	Unidentified depsides and terpenoids
**31**	*Peltigera rufescens*	0.6	Nd	Nd
**32**	*Pleurosticta acetabulum*	1.9	Norstictic acid; connorstictic acid and terpenoids	Norstictic acid Terpenoids
**33**	*Pseudevernia furfuracea*	9.8	Atranorin	Atranorin
**34**	*Ramalina fastigiata*	3.5	Evernic acid and (+)-usnic acid	Evernic and usnic acids
**35**	*Usnea florida*	5.4	Alectorialic, bourgeanic, diffractaic acids; squamatic, thamnolic and (+)-usnic acids	Usnic and thamnolic acids
**36**	*Xanthoparmelia conspersa*	4.8	Stictic acid and derivatives; (+)-usnic acid; hyposalazinic and menegazziaic acids	Stictic and usnic acids
**37**	*Xanthoparmelia tinctina*	3.3	Salazinic acid; norstictic acid; protocetraric acid and (+)-usnic acid	Salazinic and usnic acids
**38**	*Xanthoria parietina*	2.3	Parietin	Parietin

**Table 2:** Lichen extracts effective against the maturation phase of *Candida albicans* biofilms (concentration < 200 µg/mL at 24 h or 48 h). Kruskal-wallis test: *p* < 0.03, in duplicate, *n* = 2; Only extracts having demonstrated an anti-maturation activity (IC_50___mat 48 h_ ≤ 50 µg/mL) have been selected and tested against pre-formed biofilms with an age of 24 h. This anti-biofilm activity was evaluated after a 48 h incubation, Kruskal-wallis test: *p* < 0.0002, in sextuplicate *n* = 2; colony-forming unit (CFU) counts: Wilcoxon test: *p* < 0.04, in duplicate, *n* = 2; SD = standard deviation.

No.	Lichens	Anti-Maturation Activity IC_50_ (µg/mL)		Anti-Biofilm Activity	
		24 h	48 h	IC_50_ (µg/mL)	CFU Counts Inhibition (% ± SD)
**1**	*Anaptychia ciliaris*	≥200	100	-	-
**3**	*Cetraria islandica*	≥200	100	-	-
**11**	*Cladonia ramulosa*	≥200	50	50	85 ± 16
**16**	*Cladonia uncialis*	≥200	25	<10	90 ± 14
**17**	*Evernia prunastri*	25	1.56	<10	86 ± 11
**30**	*Peltigera hymenina*	50	12.5	50	80 ± 22
**31**	*Peltigera rufescens*	≥200	100	-	-
**34**	*Ramalina fastigiata*	25	3.125	<10	82 ± 17
**36**	*Xanthoparmelia conspersa*	100	25	<10	94 ± 5
**37**	*Xanthoparmelia tinctina*	≥200	6.25	100	82 ± 14
**38**	*Xanthoria parietina*	≥200	100	-	-

## Supplementary Materials

Supplementary materials are available online. Figures S1, S2 and Table S1.
