# Supplementary material for: Antifungal and Anti-Biofilm Activities of Acetone Lichen Extracts against Candida albicans
Source: Molecules. 2017 Apr 19;22(4):651. doi: 10.3390/molecules22040651 (PMC6154547; doi:10.3390/molecules22040651)
Supplement: Supplementary file 1 [file molecules-22-00651-s001.pdf]

Figure S1: HPLC chromatograms at 270 nm of extracts displaying an anti-biofilm activity.

A- *Cladonia ramulosa* acetone extract

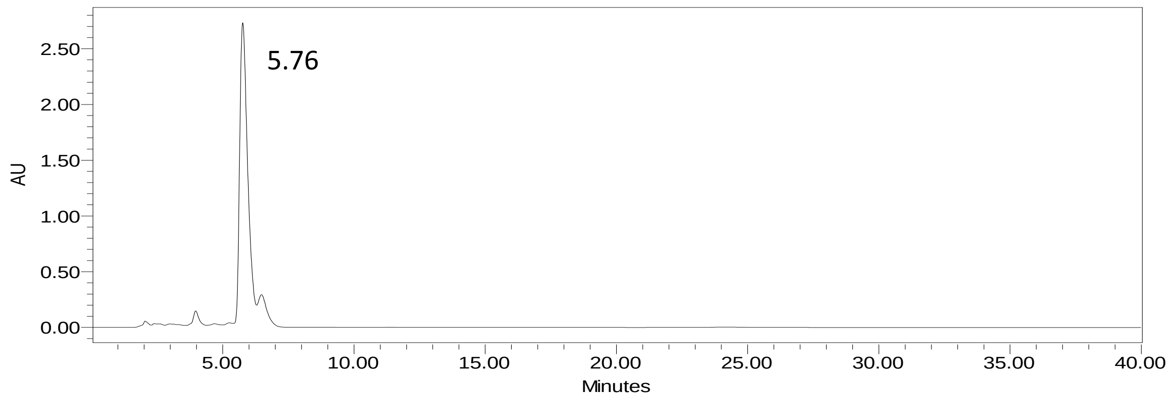

B- *Cladonia uncialis* acetone extract

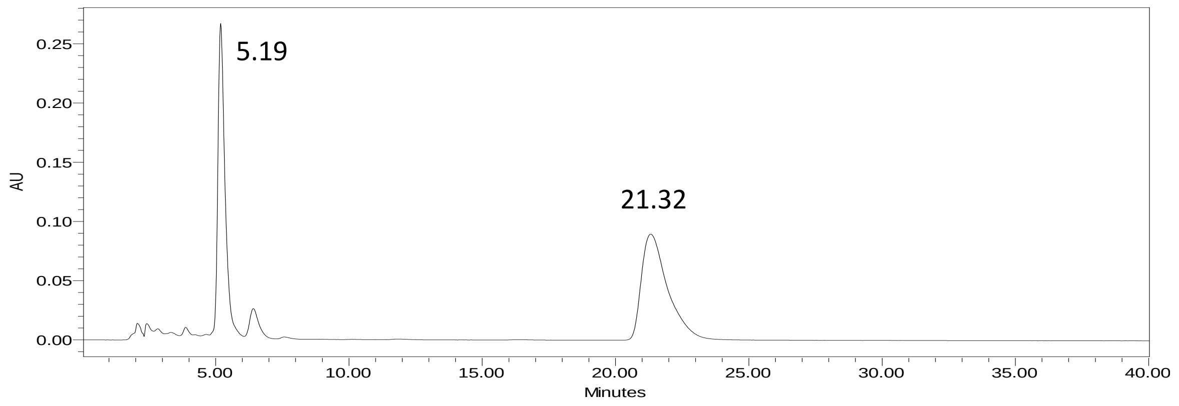

C- *Evernia prunastri* acetone extract

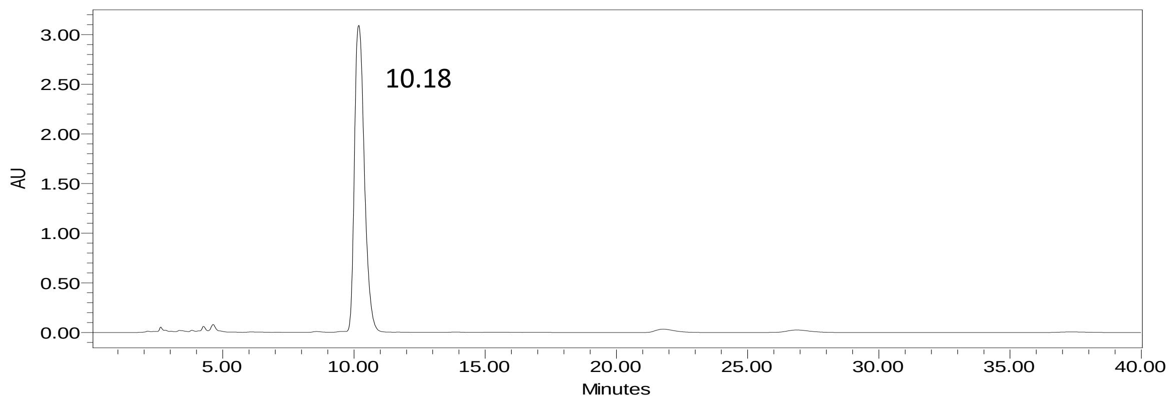

D- Xanthoparmelia conspersa acetone extract

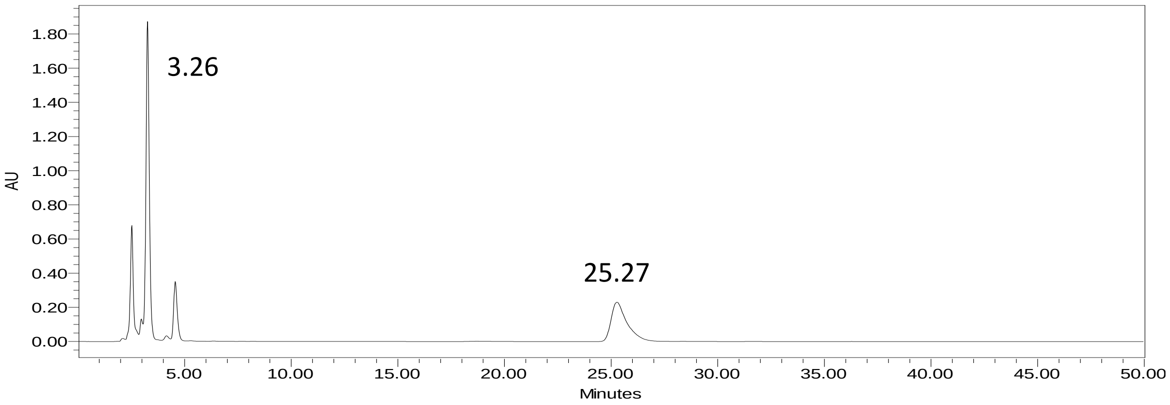

E- Xanthoparmelia tinctina acetone extract

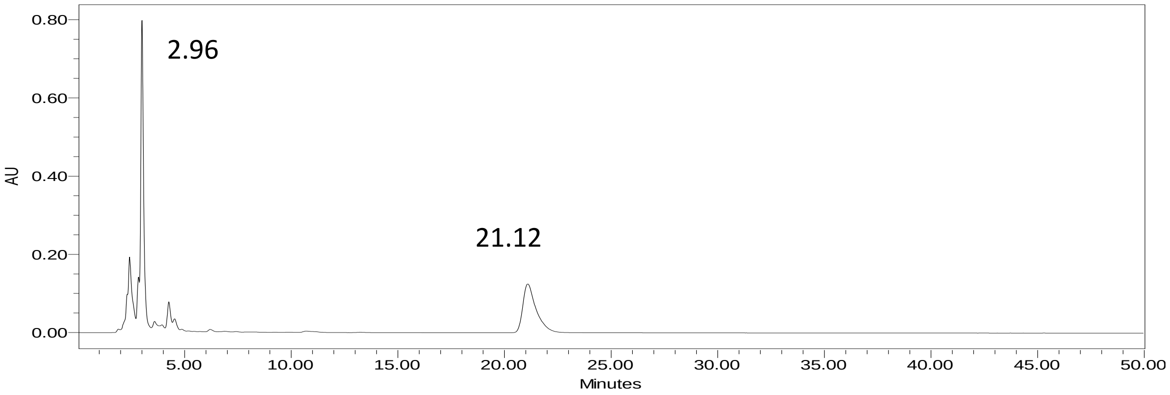

F- Peltigera hymenina acetone extract

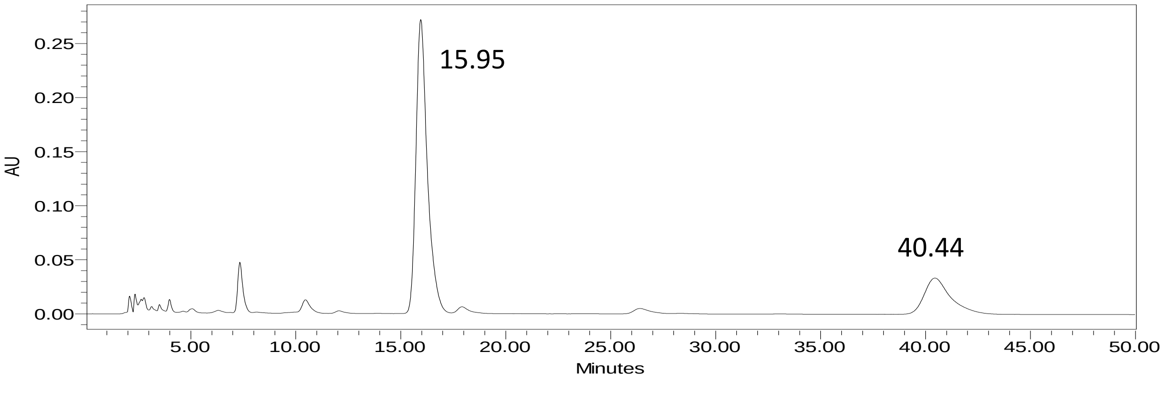

G- Ramalina fastigiata acetone extract

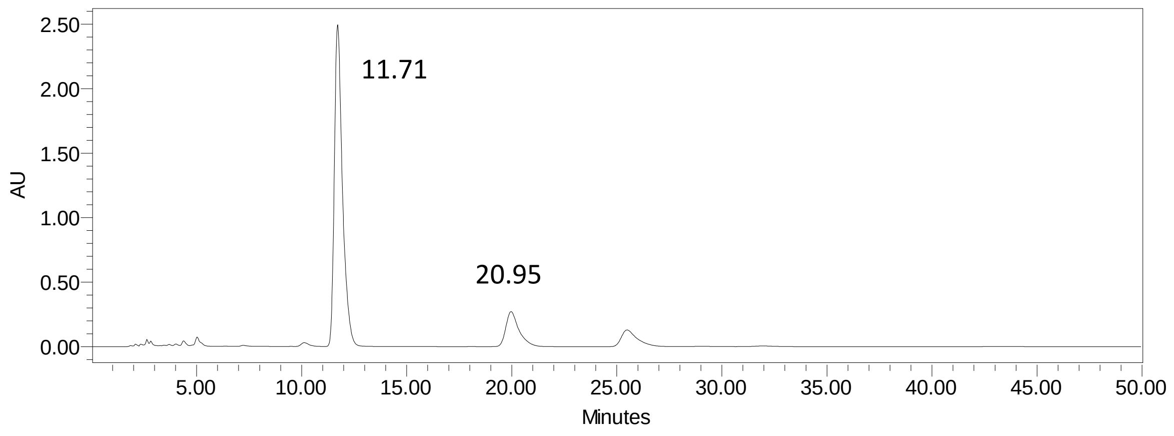

Experimental – Supplementary Information

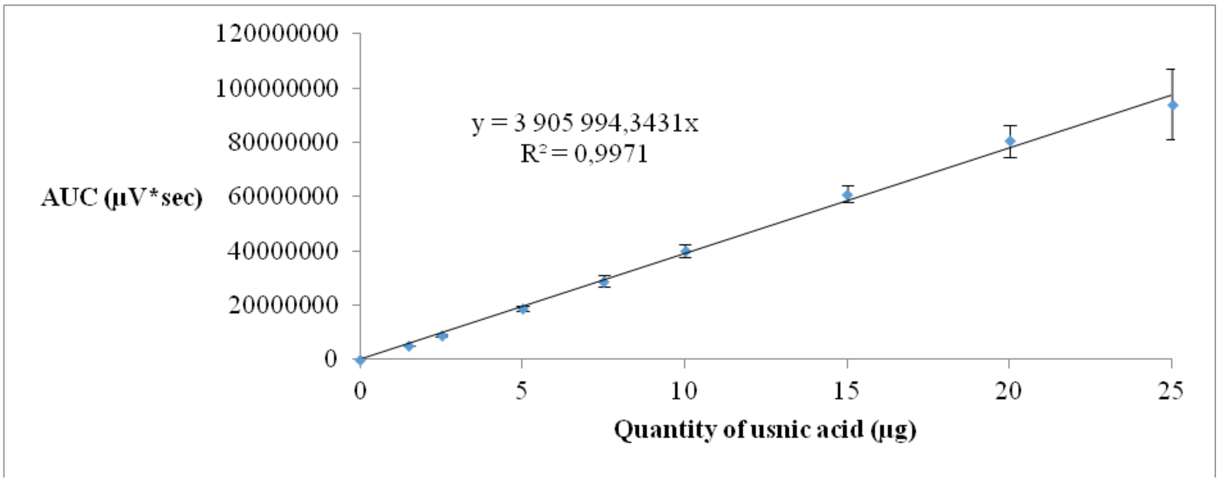

Figure S2: Usnic acid dosage by HPLC.

A calibration curve using usnic acid has been prepared in acetone in order to obtain concentrations ranging between 0.15 and 2.5 mg/mL. Ten microliters of the standard solution have been injected in triplicate.

Extracts have been solubilized in acetone in order to obtain a concentration of 1 mg/mL and 10µL have been injected. The area under the curve (AUC) has been taking to calculate the quantity of usnic acid in the *Usnea florida* and *Flavoparmelia caperata* extracts.

Table S1: Voucher reference number, date and place of collection of lichens.

Extract number	Lichens	Reference Herbarium	Date of collection	Place for collect City (Department)
**1**	*Anaptychia ciliaris*	HL-L04/14-01	05/04/2014	Cieux (87)
**2**	*Bryoria fuscesens*	HL-L10/09-10	06/10/2009	Peyrol (87)
**3**	*Cetraria islandica*	HL-L08/12-03	15/08/2012	L’Echo (73)
**4**	*Cetrelia olivetorum*	HL-L10/09-02	06/10/2009	Gouron-Murat (19)
**5**	*Cladonia fimbriata*	HL-L04/14-03	05/04/2014	Le Chatenet (87)
**6**	*Cladonia furcata*	HL-L10/11-29	17/10/2011	Les Roumilloux (19)
**7**	*Cladonia glauca*	HL-L10/11-08	20/10/2011	St Angel (19)
**8**	*Cladonia gracilis*	HL-L10/11-06	03/10/2011	Bussière-Boffy (87)
**9**	*Cladonia incrassata*	HL-L09/11-02	06/09/2011	Verneuil/Vienne (87)
**10**	*Cladonia parasitica*	HL-L09/11-15	15/09/2011	Verneuil/Vienne (87)
**11**	*Cladonia ramulosa*	HL-L10/11-03	03/10/2011	Bussière-Boffy (87)
**12**	*Cladonia rangiferina*	HL-L10/11-13	17/10/2011	Les Roumilloux (19)
**13**	*Cladonia scabriuscula*	HL-L10/11-02	03/10/2011	Landes de Frochet (87)
**14**	*Cladonia squamosa*	HL-L09/11-16	15/09/2011	Verneuil/Vienne (87)
**15**	*Cladonia subulata*	HL-L03/14-01	04/03/2014	Limoges (87)
**16**	*Cladonia uncialis*	HL-L10/11-11	18/10/2011	Soudeilles (19)
**17**	*Evernia prunastri*	HL-L01/14-01	05/01/2014	Verneuil/Vienne (87)
**18**	*Flavoparmelia caperata*	HL-L10/11-01	18/10/2011	Soudeilles (19)
**19**	*Hypogymnia physodes*	HL-L10/09-07	05/10/2009	Serandon (19)
**20**	*Lasallia pustullata*	HL-L10/11-09	03/10/2011	Bussière-Boffy (87)
**21**	*Lepraria membranacea*	HL-L10/11-12	17/10/2011	Les Rouchilloux (19)
**22**	*Leprocaulon microscopicum*	HL-L10/09-05	05/10/2009	Sérandon (19)
**23**	*Neofuscellia pulla*	HL-L04/14-12	07/04/2014	Limoges (87)
**24**	*Nephroma parile*	HL-L12/09-02a	06/12/2009	Masléon (87)
**25**	*Parmelia saxatilis*	HL-L07/10-02	12/07/2010	Crozat (87)
**26**	*Parmelia sulcata*	HL-L04/14-10	07/04/2014	Limoges (87)
**27**	*Platismatia glauca*	HL-L10/09-11	06/10/2009	Gourdon-Murat (87)
**28**	*Pleurosticta acetabulum*	HL-L10/11-26	20/11/2011	Les Ulis (91)
**29**	*Peltigera collina*	HL L10/14-01	03/10/2014	Soudeilles (19)
**30**	*Peltigera horizontalis*	HL-L05/14-02	10/05/2014	Largillat (25)
**31**	*Peltigera hymenina*	HL-L10/11-25	20/10/2011	St Angel (19)
**32**	*Peltigera rufescens*	HL-L06/12-01	24/06/2012	La Roque Gageac (24)
**33**	*Peseudevernia furfuracea*	HL-L05/14-01	10/05/2014	Largillat (25)
**34**	*Ramalina fastigiata*	HL-L04/14-09	05/04/2014	Cieux (87)
**35**	*Usnea florida*	HL-L10/11-31	19/10/2011	St Merd les Oussines (19)
**36**	*Xanthoparmelia conspersa*	HL-L04/14-11	07/04/2014	Limoges (87)
**37**	*Xanthoparmelia tinctina*	HL-L04/14-13	07/04/2014	Limoges (87)
**38**	*Xanthoria parietina*	HL-L03/14-04	14/03/2014	Limoges (87)
